# Using the theoretical domains framework to identify barriers and enabling factors to implementation of guidance for the diagnosis and management of nonalcoholic fatty liver disease: a qualitative study

**DOI:** 10.1093/tbm/ibz080

**Published:** 2019-05-23

**Authors:** Kate Hallsworth, Stephan U Dombrowski, Stuart McPherson, Quentin M Anstee, Leah Avery

**Affiliations:** 1 Institute of Cellular Medicine, Faculty of Medical Sciences, Newcastle University, Newcastle upon Tyne, UK; 2 Liver Unit, Newcastle Upon Tyne Hospitals NHS Foundation Trust, Newcastle Upon Tyne, UK; 3 Faculty of Kinesiology, University of New Brunswick, Fredericton, NB, Canada; 4 Centre for Rehabilitation, Exercise and Sports Science, Teesside University, Tees Valley, UK

**Keywords:** Nonalcoholic fatty liver disease (NAFLD), Nonalcoholic steatohepatitis (NASH), Type 2 diabetes (T2D), Theoretical Domains Framework (TDF), Qualitative interviews, Guideline implementation

## Abstract

Nonalcoholic fatty liver disease (NAFLD) is the most common liver condition worldwide and is steadily on the increase. In response, national and international guidance have been developed to standardize diagnosis and guide management of the condition. However, research has highlighted a discordance between published guidance and clinical practice. The purpose of this study is to identify barriers and enabling factors to implementation of guidance to inform the development of an intervention. We interviewed 21 health care professionals and 12 patients with NAFLD. Topic guides were developed with reference to national and international guidance. Data were content analyzed using the Theoretical Domains Framework. Beliefs about consequences and professional role and identity were the most prominent domains identified from health care professionals in the context of diagnosis and management of NAFLD. Environmental context and resources, memory, attention and decision processes, goals, behavioral regulation, knowledge, and skills emerged as important barriers/facilitators to implementation of guidance targeting management of NAFLD. Knowledge and beliefs about consequences were the most prominent domains from the perspective of patients. Social influences, environmental context and resources and behavioral regulation were most prominent in the context of NAFLD management. Guideline implementation can be improved by use of interventions that target standardized use of diagnostic criteria by health care professionals. Training of health care professionals was identified as important to improve care delivered to patients in order to effectively manage NAFLD. Interventions that target knowledge of patients, in particular, raising awareness that NAFLD can be progressive when not actively managed would facilitate implementation of guidance.

## Teaser

Healthcare professionals and patients highlighted a need to increase knowledge about what NAFLD is and how to manage it through lifestyle behaviour change.

**Implications**

**Practice:** Evidence-based training for clinicians should be systematically developed to address identified barriers and facilitators to support guideline implementation and subsequent patient behavior change.

**Policy:** Policymakers should support the development and promotion of systematically developed evidence-based training for clinicians to facilitate implementation of guidelines.

**Research:** Future research should aim to identify optimal behavioral strategies to target the identified barriers and facilitators with a view to inform the content of training to facilitate effective implementation of NAFLD guidelines.

## BACKGROUND

Nonalcoholic fatty liver disease (NAFLD) is the most common liver condition worldwide and is largely associated with dietary excess, inactivity, and being overweight. Its prevalence is estimated to be 20%–30% of the adult population [1,2] and this increases substantially in people with Type 2 diabetes mellitus (T2DM) or those with multiple features of the metabolic syndrome [3]. In the absence of specific approved pharmaceutical agents for NAFLD, changes to diet and increases in physical activity/exercise to achieve weight loss is the principal therapeutic recommendation [4,5]. Evidence supporting the use of lifestyle interventions is strong and has shown clinically significant reductions in liver fat and improvements in glucose control/insulin sensitivity in those with NAFLD [6–17]. Liver inflammation and fibrosis can also be improved/reversed with a weight loss of ≥7%–10%, and research reports a dose–response relationship between weight loss percentage and overall histological changes, with the greatest improvements in liver health observed in individuals who achieve the greatest weight loss [17].

In response to the evidence on the effectiveness of lifestyle interventions for the management of NAFLD, the European Clinical Practice Guidelines [18] and the National Institute for Health and Clinical Excellence (NICE) Guidelines [19] were published in 2016 followed by the American Association for the Study of Liver Disease Guidelines published in 2018 [20]. All highlight the importance of lifestyle behavior change in all patients with NAFLD regardless of disease severity. However, despite the publication of these guidelines, a gap remains between recommended clinical care behaviors and actual care delivery [21]. Specifically, there are inconsistencies in the way in which patients are diagnosed. For example, different tools are used to make a diagnosis and in some cases validated tools are not used at all. This often leads to inappropriate referrals to secondary and tertiary care. In terms of NAFLD management, the majority of patients are monitored for disease progression on an annual basis but not actively managed—that is, patients are rarely given the information and support they require to make lifestyle behavior changes.

In terms of NAFLD diagnosis, national and international guidelines [18–20] suggest that when a NAFLD diagnosis is considered likely and based on the patient’s lifestyle and medical history, disease severity should be assessed. In the first instance, a noninvasive validated tool such as the NAFLD Fibrosis Score or FIB4 [22,23] is recommended. If there is a doubt regarding the diagnosis or if the patient is triaged to be at an indeterminate/high risk of advanced disease, they should be referred to a specialist physician in secondary/tertiary care (usually a hepatologist or gastroenterologist) for further investigation. At all points in this care pathway, patients could benefit from lifestyle intervention regardless of disease severity.

Targeting NAFLD with lifestyle behavior change is essential to improve patient health, particularly as excess liver fat is an independent risk factor for the development of T2DM and cardiovascular disease [3]. Despite the accumulating evidence supporting the use of lifestyle interventions for the management of NAFLD [5], currently there is no defined clinical lifestyle pathway [24]. This would involve the provision of evidence-based lifestyle behavior change intervention and support imbedded in to clinical practice as a referral pathway or as part of routine consultations.

This aim of this qualitative study was to identify barriers and enabling factors to implementation of guidance for the diagnosis and management of NAFLD. Specifically, we aimed to explore with health care professionals ways in which the diagnostic process could be improved in order to ensure patients are appropriately referred and to identify how patients could be best supported to make lifestyle behavior changes. We also obtained patient views on how to improve the diagnostic process and subsequent management of NAFLD. Obtaining the views of both health care professionals and patients was considered important to establish which areas should be the focus of intervention that meets the needs of both groups.

The guideline-recommended practice behaviors of interest were the diagnosis of NAFLD, referral of patients following diagnosis, and management of NAFLD (i.e., targeting diet and physical activity behaviors of patients to initiate weight loss).

## METHODS

This study was approved by the NHS London-Riverside Research Ethics Committee (REC reference: 15/LO/0815). Informed written consent was obtained from health care professionals and patients by a member of the research team prior to the conduct of the study. Patients were reimbursed costs for travel to the Clinical Research Facility where interviews took place.

Three members of the research team have received formal academic training in qualitative research methods. Two members of the team are health psychologists who are experienced in the conduct of qualitative research, specifically in the context of intervention development and implementation.

## DESIGN AND SETTING

We conducted semi structured interviews with health care professionals, including hepatologists, gastroenterologists, diabetologists, practice nurses, general practitioners, and patients with NAFLD across primary, secondary, and tertiary care settings in Newcastle upon Tyne, UK.

## INTERVIEW TOPIC GUIDES

Two topic guides (one for health care professionals and one for patients) were used to conduct the interviews and each was developed with reference to national [19] and international guidelines [18] for the diagnosis and management of NAFLD. Each topic guide included open ended questions to elicit perceptions on barriers and facilitators to guideline implementation (see Supplementary Materials 1 and 2).

## PARTICIPANTS

We employed a purposive sampling strategy, maximal variation [25] in order to identify shared patterns in the data generated from health care professionals and patients.

### Health care professionals

We recruited health care professionals from specialties including hepatology, gastroenterology, diabetology, and general practice to gain a range of perspectives. These clinical specialties were chosen as health care professionals were likely to see patients with NAFLD on a regular basis. It was also considered important to obtain the views from both hospital and community-based clinicians working across specialist and generalist services. As such, health care professionals from 2 NHS Hospitals Trusts and 11 UK NHS clinical commissioning groups were invited to take part in the study. Invitations were sent via email or by verbal invitation. Health care professionals were interviewed by a member of the research team.

### Patients

We recruited a sample of adults aged ≥18 years with a diagnosis of NAFLD identified by review of medical records by primary and secondary care teams. Patients were invited to take part in the study by letter. We aimed to recruit a sample of patients taking in to account age, gender, length of time since diagnosis and those who had attended appointments in primary and/or secondary/tertiary care settings. Those who were interested in taking part were asked to contact the research team directly to arrange an interview. The research team did not have any influence on patient recruitment. Patients were subsequently interviewed by one member of the research team.

## METHODS TO MAXIMIZE TRUSTWORTHINESS OF DATA

A number of established methods were used to maximize the trustworthiness of the data generated and subsequent themes reported. These included triangulation of data sources (i.e., interviews with primary and secondary health care professionals and patients) and analysts (i.e., data were independently coded by two researchers) to enhance credibility and provision of a thick description to add context supported by direct quotes to enhance transferability. Dependability and confirmability were enhanced by the development of a coding system and transparent reporting of the conduct of the study, including data analyses and interpretation [26].

## ANALYSIS

Data generated from interviews were analyzed using the Theoretical Domains Framework (TDF) [27]. The TDF was developed to simplify and integrate 33 behavior change theories and 128 key theoretical constructs related to behavior change. These were synthesized into a single framework to assess implementation and clinical behaviors around evidence-based guidelines and therefore, appropriate for use in the current qualitative study. The TDF originally comprised of 12 domains, which was subsequently validated and refined to 14 domains. These are knowledge, skills, social/professional role and identity, beliefs about capabilities, optimism, beliefs about consequences, reinforcement, intentions, goals, memory, attention, and decision processes, environmental context and resources, social influences, emotion, and behavioral regulation. The 14 domain framework was used for the purpose of this study.

All interviews were audio-recorded and transcribed verbatim. A three-stage process was followed in order to analyze interview transcripts of both health care professionals and patients. Firstly, two interview transcripts (one health care professional and one patient transcript) were pilot coded independently by two researchers to agree a coding strategy (i.e., to ensure both researchers were coding consistently and to discuss and resolve any difficulties when applying the TDF). Initial findings of the two pilot transcripts were discussed before coding the remaining transcripts. Second, data from the remaining transcripts were independently coded by the same two researchers and this involved reading and rereading transcripts, coding the content into themes and subthemes, and mapping these, with supporting direct quotes, to an appropriate theoretical domain of the TDF [27] (see Tables 1 and 2). Although the TDF was used as a coding framework, code generation outside of the TDF was possible to ensure all data generated were coded and reported. Finally, a discussion took place to agree the most prominent domains as barriers and facilitators to implementation of guidance. Judging the most prominent domains is customary within TDF guided analyses [28] in order to provide suggestions for which domains should be target for intervention. These were identified based upon the following criteria: (a) the frequency in which specific views or beliefs within each domain were expressed by participants and (b) the strength of views or beliefs within each domain that were discussed at great length. Illustrative quotes were used to support domains and subthemes within domains.

**Table 1:** Barriers and facilitators to diagnosis and management of nonalcoholic fatty liver disease (NAFLD) in the context of guideline implementation from the perspective of health care professionals

Theme/subtheme	Illustrative quotation	Theoretical domain(s) assigned to subtheme
1.0 Diagnosis of NAFLD		
1.1 Subtheme: Local guidelines have improved the diagnostic process	“Guidelines are now more widely used, actually we get quite a lot that come [to Secondary Care] with a NAFLD Fibrosis Score already calculated and have done all the tests and then it’s a just liver biopsy. So the new guidelines have made a big difference. Not everyone’s using them yet but I think if we give it a couple of years, simple intervention will have made a huge difference”	Optimism
1.2 Subtheme: Inconsistent use of diagnostic criteria in primary care leads to variability in the appropriateness of referrals	“Very few (patients) that I’ll see once, discharge and say, this is a waste of everybody’s time… probably the GPs are actually filtering out a lot of the ones that are thought to be just simple steatosis…maybe the GPs are looking after lots of people that we might want to get hold of and might want to stage the disease properly. So maybe they’re only referring in the ones that they’re most worried about and there’s another cohort that we’re never seeing…” “The risk of NAFLD is recognised in the community. The NAFLD fibrosis score is calculated and then for example, patients with indeterminate or high scores are passed on to Secondary and Tertiary Care. The reality is that in some practices that is exactly what happens, which is excellent. In other practices, it is completely ad-hoc. Patients may be referred on with the most minor changes in liver biochemistry. And similarly, patients with more significant changes may not be referred on”	Beliefs about consequences
1.3 Subtheme: Patients have little or no understanding of their diagnosis	[patients are] “told they have a problem with their liver [by a GP]. Many of them, unless it’s been properly explained to them by the GP are puzzled why that is because they associate liver disease with alcohol consumption” “We’ve got a population of people…that I don’t think know that they’ve got this diagnosis… historically, it’s not really been something we’ve been proactive about doing anything with..”	Beliefs about consequences
1.4 Subtheme: Diagnosis of NAFLD may initiate anxiety in patients	“People who really didn’t want to know about it or hear about it [NAFLD diagnosis], and other people obviously get quite anxious about it. And I suspect sometimes we maybe play the condition down a little bit…” “I think that a proportion of these people are worried that they have got cancer, and that is why they have sought, you know, depending on why they were originally had investigations, they still anxious to seek reassurance”	Beliefs about consequences
2.0 Management of NAFLD		
2.1 Subtheme: Referring to local guidelines inform decision-making about management approach	“if somebody has abnormal LFTs…refer to the (local) guidelines at about what level you should then go on to refer and what level you would just monitor in general practice…need to be on the lookout that they’re not going to develop further specific liver problems”	Memory, attention, and decision processes
2.2 Subtheme: Monitoring of the condition is more likely than active management	“I would see them more with a chronic liver disease slant. I don’t think I would ever enter into the situation where I’m ever actually managing their weight loss or fatty…Yes, I wouldn’t ever…I’m not sure I could really afford to get too involved in the, kind of, active management of risk factors and stuff. I would definitely shun that back to primary care or to the patients themselves to be honest. I’m quite keen on getting the patient to take the responsibility” *“*So if you score as high risk or if your biopsy shows that you’ve got fibrosis then you’re somebody that we’re going to monitor for complications of cirrhosis. So those people will go into…we’ll keep hold of them for a six-monthly review and they’ll get the HCC surveillance so they’ll get their ultrasound six-monthly…They’ll get endoscopies at appropriate intervals”	Professional role and identity
2.3 Subtheme: Training is required to improve knowledge, diagnosis, and management of NAFLD	“Increasing education of GPs is the primary thing. If we can get them to follow the guidelines we’ve published, actually we are 95% of the way there, because they will recognise abnormal LFTs and they will start to do the right things and intervene or whatever and identify the sick patient” “Probably under treat and under monitor most of these people. We’re very aware that there’s lots and lots of people have mildly abnormal liver function tests that we never really go into great detail, as long as it’s stable. So I think there probably is a training need there to know who it is we should be looking at and when we should be referring them on” “The problem with NAFLD is diagnosing it and I think the lack of treatment specific for NAFLD is the biggest problem…we’re still coming back to telling them to exercise and lose weight…” “I think the lack of understanding in primary care is very evident sometimes and a lot of them [patients] come very angry because they’ve been accused of drinking alcohol” “I think it [increased knowledge] would be beneficial the fact that when they start to ask me questions at least possibly I could give them an answer rather than saying, you need to speak to the GP about that one”	Knowledge Skills
2.4 Subtheme: Training to effectively target lifestyle behavior change is required	“in terms of assessing weight loss readiness, there could be a little bit more work done with that, prior to, um, the patients working with me in the clinic…good identification of those [patients] that are ready to make those changes…makes a huge difference to your outcomes in your care plan and in your work” “Skills of motivational interviewing and behaviour change are probably where I think there is still an awful lot of people telling people what to do. And that culture needs to change” “I think if you were looking for what little things could make a difference within a much more limited budget then having some form of training on intervention, motivational interviewing would be really helpful. Because actually if you can improve the skills of the people who are seeing the patients then it’s more helpful than doing nothing and it’s less reliant on… Someone else doing it. And I think it probably would help if you’re referring to something like an exercise programme you have to have got somebody on board with that, don’t you?”	Beliefs about consequences Skills
2.5 Subtheme: A multidisciplinary team with the necessary expertise is required to successfully implement guidance on the management of NAFLD	NAFLD service “very focused on one aspect of lifestyle…we don’t have anyone who’s specialised in giving physical activity…a good proportion of patients who just don’t know or don’t know what to do or don’t know how they can adapt certain things and, yes, so that would be a major part of the clinic that’s missing” “Multi-disciplinary team…Dietetics…key to delivery. Work on lifestyle change…individuals who develop NAFLD…are not particularly open to increasing exercise…individuals with fatty liver lack the confidence to make these changes…giving some advice and enabling people to make those changes would be useful…psychological support…not uniformly available…” “What I ideally wanted was almost like a one-stop shop… I can foresee a great big clinic with me, the physio, a dietician, all doing a one-stop appointment for them to go out”	Beliefs about consequences Environmental context and resources Skills
2.6 Subtheme: Tools and resources are needed to support management of NAFLD	“Food diaries, pedometers to set people simple goals…to nudge people towards slightly greater exercise and nudge people slightly lower calorific intakes. It doesn’t have to be traumatic, in fact the less traumatic it is the easier it will be to sustain it” “online thing where patients can, kind of, log in, they can track their progress, there is loads of advice about exercise, diet, psychology, all that sort of thing…pedometers…HR monitors…Fitbits” “a simple patient information leaflet” “Some kind of liver with fat in it and inflammation on it and scarring so I could actually say, “This is what a liver should look like and this is what happens to your liver.”	Behavioral regulation Environmental context and resources Goals
2.7 Subtheme: There is no treatment for NAFLD other than lifestyle advice	“I mean that’s the trouble with non-alcoholic fatty liver disease. Apart from lifestyle, there’s not a lot else to do [treatment wise]...” “normally you just tell them to lose 10% of their weight and that’s it. There’s no treatments”	Beliefs about consequences
2.8 Subtheme: Lack of awareness of external lifestyle services	“Not really sure what’s involved (in making a referral to other lifestyle services)…It’s not something I’m aware of being available…if it is that would be good.” “I genuinely don’t know where to send them…go and speak to the GP because they’ll have more knowledge than I have”	Knowledge
2.9 Subtheme: The option to refer to an external lifestyle service would facilitate management of patients with NAFLD	“What has grabbed me most is the idea of being able to prescribe interventions, and order up pedometers… I would like to be able to send someone to a service…what I would like, is to be able to pass the patient on to some sort of lifestyle coach, and then for the next time I see them to have more data, so that I can look at what their calorie intake, and what their eating habits is, what their pedometer shows, what their self-filled questionnaire about their self-efficacy….” “Pretty much every patient I see could do with some sort of lifestyle coaching of some description, tailored to them, be it alcohol or weight management or IBS or, you know…” “fantastic to be able to send them to something in the community”	Environmental context and resources

Given the explicit nature of the TDF as the guide for coding, all interview transcripts were coded and analyzed by hand and no qualitative software was required. In line with published guidance, interview transcripts were analyzed until the point of data saturation—that is, interviews with health care professionals and patients ceased once data saturation had been reached. Data saturation was considered for health care professionals and patients separately—that is, no further interviews were conducted within each of these groups once data saturation had been reached. Data saturation was assumed when subsequent interviews did not lead to the identification of additional barriers and facilitators or differing views on previously identified barriers and facilitators [29].

## RESULTS

Twenty-one health care professionals (10 male; 11 female) were recruited from primary (*n* = 7) and secondary/tertiary care (*n* = 14) settings. Eleven were consultants specializing in hepatology (*n* = 4), gastroenterology (*n* = 4), and diabetology (*n* = 3); six were primary care physicians; two were dieticians; one a hepatology specialist nurse; and one a primary care practice nurse.

Twelve patients (8 male; 4 female; aged 58.9 years [range 44–72 years]) were recruited from primary (*n* = 8) and secondary/tertiary care settings (*n* = 4). The average time since diagnosis was 3.9 years (range: 1 month to 19 years). Two of the eight patients recruited from primary care had also attended appointments in secondary/tertiary care. Interviews with health care professionals lasted approximately 20 min (range: 7–32 min) and patient interviews approximately 10 min (range: 5–17 min).

## HEALTH CARE PROFESSIONAL PERSPECTIVES

Nine theoretical domains were identified in relation to barriers and facilitators to guideline implementation from the perspective of health care professionals. In terms of NAFLD diagnosis, optimism and beliefs about consequences were identified. In terms of NAFLD management, beliefs about consequences, memory, attention, and decision processes, professional role and identity, knowledge, skills, goals, behavioral regulation and environmental context and resources were identified (see Table 1). The most prominent domain identified for both diagnosis and management of NAFLD was beliefs about consequences.

## NAFLD DIAGNOSIS AND REFERRAL

### Optimism

Health care professionals felt that the introduction of local guidance for the diagnosis of NAFLD had worked well to increase the number of appropriate referrals from primary to secondary/tertiary care (i.e., patients referred did on the whole require secondary/tertiary care specialist input). “Guidelines are now more widely used, actually we get quite a lot that come [to Secondary Care] with a NAFLD Fibrosis Score already calculated and have done all the tests…. So the new guidelines have made a big difference.” It was also believed that primary care professionals may only be referring patients they are most concerned about or those whose condition had progressed from mild NAFLD to a more serious form of liver disease (i.e., nonalcoholic steatohepatitis [NASH] with significant fibrosis). Although the implementation of local guidance was considered beneficial for diagnosing patients, variation in guideline adherence was noted and that some inappropriate referrals remained. It was, therefore, reported that greater awareness was required around the need to use validated tools to diagnose and standardized training on how to use them. It was also considered important to raise awareness of when it is appropriate to refer to secondary/tertiary care (e.g., when more serious forms of NAFLD are diagnosed and require specialist input). Overall, health care professionals were optimistic that with appropriate training on the use of guidance and awareness raising, the diagnostic process could be improved.

### Beliefs about consequences

Following diagnosis of NAFLD in primary care, secondary health care professionals reported patients having very little or no understanding of their diagnosis when attending secondary care appointments. The majority indicated that information should be provided at the time of diagnosis. However, some primary health care professionals believed that providing information at this time could lead to an increase in anxiety in patients because some “do not want to know about it or hear about it, and other people get quite anxious about it.” Therefore, NAFLD was regularly “played down” by health care professionals.

## NAFLD MANAGEMENT

### Beliefs about consequences

Health care professionals reported providing advice to patients to lose weight and exercise more in order to manage their NAFLD but emphasized that patients often did not follow this advice—that is, it was believed that time spent providing lifestyle advice would not be worthwhile. However, it was acknowledged that patients lacking knowledge about their condition was one possible explanation for why advice was not acted upon.

### Professional role and identity

In terms of NAFLD management, there was a general consensus among health care professionals that the condition was actively monitored rather than managed (i.e., patients were seen usually on an annual basis where they would undergo a series of tests to assess disease stage and progression). “I do not think I would ever enter into the situation where I’m ever actually managing their weight loss or fatty [liver].” The belief was that it was not the role of the specialist to target lifestyle behavior change, although some reported providing advice to lose weight.

### Environmental context and resources

Health care professionals reported that limited time during consultations meant that lifestyle behavior change could not be fully addressed, particularly when patients lacked knowledge about what NAFLD is and how it can be managed. In addition, lack of available lifestyle support resources within clinics and external services for referring patients to meant that health care professionals were restricted in terms of the extent to which they could adequately target lifestyle behavior change. It was felt that greater awareness of local lifestyle services was required so that patients could be referred for support outside of the clinical setting: it would be “fantastic to be able to send them to something in the community.”

### Knowledge

Where services to refer patients to were not available, it was reported that knowledge and skills of the clinical team should be targeted with training. It was highlighted that some primary health care professionals lacked specific knowledge about NAFLD and reported difficulties in being able to communicate to patients what it is, the risks associated with it, and how it can be managed. It was also felt that the clinical team lacked knowledge and skills in lifestyle behavior change or, where this expertise did exist (e.g., in secondary care it was reported that a member of the clinical team did have expertise in this area), it was not feasible for one individual within a team to manage the large number of patients being referred. It was emphasized that knowledge and expertise in the context of lifestyle behavior change within the clinical team was required to offer a multidisciplinary team approach NAFLD management.

### Skills

The majority of health care professionals felt that they were not adequately trained to effectively target lifestyle behavior change themselves and as such suggested that training in this area would be beneficial, “I think if you were looking for what little things could make a difference within a much more limited budget then having some form of training on intervention, motivational interviewing would be really helpful.”

### Goals

Health care professionals felt that it was important to set patients goals as a means of “nudging” them toward increased levels of physical activity and exercise and to reduce calorie consumption. It was felt that graded goals would be most effective to ensure that the changes were realistic and could be sustained in the long term.

### Behavioral regulation

A number of health care professionals suggested that “food diaries, [and] pedometers to set people simple goals…to nudge people towards slightly greater exercise and nudge people slightly lower calorific intakes” would be useful. This would allow patients to track their progress against dietary and activity goals in order to change their lifestyle behaviors. Other suggestions included an online programme containing information and advice about diet, exercise, and the psychological aspects of making lifestyle changes. Others suggested new ways of communicating to patients about what their liver looks like compared to how it should look using models to help regulate behavior. All agreed that monitoring of lifestyle behaviors was important.

### Memory, attention, and decision processes

Health care professionals reported management of patients with NAFLD to involve monitoring rather than active lifestyle management and that the decision-making process was informed by local guidance. For example, if a patient had an abnormal liver function test, primary health care professionals reported using the guidance to make a decision on whether to refer to secondary care. Whereas secondary care professionals reported referring to the guidance to make decisions on referring patients back to primary care for monitoring.

### Patient perspectives

Four theoretical domains were identified in the context of guideline implementation from the perspective of patients. These were knowledge, beliefs about consequences, social influences, and behavioral regulation (see Table 2). Knowledge and beliefs about consequences were the most prominent domains identified in relation to diagnosis and management of NAFLD.

**Table 2:** Barriers and facilitators to guideline implementation from the perspective of patients

Theme/subtheme	Illustrative quotation	Theoretical domain(s) assigned to subtheme
1.0 Diagnosis of NAFLD		
1.1 Subtheme: Diagnosis of NAFLD was unexpected	“It was only when I went for a visit, routinely, to the GP, for something completely different, that she said, ‘We have discovered that you have got this, and we need to do a blood test’. Did the blood test, and then she said, ‘I am going to refer you’, which I was quite shocked at, because I wasn’t expecting anything to become of it. Because it had been quite a while. And then she referred me to [the hospital], for a liver biopsy.”	Beliefs about consequences
1.2 Subtheme: Information provision following diagnosis of NAFLD is lacking	“I couldn’t really go into it. It was so brief, what I got off my GP. And I haven’t done much research into it myself. She did tell me I could Google it [NAFLD] and read up about it…But I haven’t.” “They tell you very little really. You know, you just get told that you’ve got fatty liver disease, but they’ll say a lot of people have fatty liver disease, it’s nothing to worry about – lots of people live all of their lives, well, most of their lives with fatty liver disease and that’s it.” “I would have liked for it to have been explained how or why you get it, because they don’t really…by what I have read sometimes it’s your diet and things like that. And well, just what you should do really, just anything…it would be nice to have a leaflet just for it to explain, and things that would help.”	Knowledge
2.0 Management of NAFLD		
2.1 Subtheme: NAFLD is monitored but not actively managed	“It’s just a matter of monitoring how you get on. Making sure you’re doing what she’s telling you to do. The next step would be a consultant, but wouldn’t they give you the same sort of information?” “I had the biopsy, and got the results back from the biopsy to say that they didn’t need to see me again – but no help, no advice, no: ‘Okay, you are at the early stages…this is what you need to do so that you don’t progress, nothing’.”	Beliefs about consequences
2.2 Subtheme: Support to make lifestyle changes to manage NAFLD is lacking	“The only thing they said was to try and sort of lose a bit of weight…But, apart from that, no, I’ve never ever had any advice or anything else.” “At the time I thought, right okay, does that mean I’m overweight or something? And then he said, “Oh you must drink a lot?”. And I went, “No, I don’t drink at all”. They said, oh right. And that was it. So they marked that down and that was the end of that really. He said, “Are you sure you don’t drink?”, I went, “No, I don’t drink at all”. [Laughter]. Can drink cause that?”	Knowledge Beliefs about consequences
3.2 Subtheme: Support from clinicians and other patients to target lifestyle behavior change would be beneficial	“Some type of intervention in terms of weight loss and dieting might be quite useful. And certainly to kind of motivate them to do it regularly. You could have just a kind of nurse in-between seeing the doctors in the hospital. Or you could take it into the community if there are so many people who’ve got non-alcoholic fatty liver disease, and develop kind of satellite clinics, for which you don’t really need a doctor.” “I think people work well in groups and support each other, and it is nice to hear other peoples’ experiences, I think that group session would be great.” “Yes, yes, like even if it was just monthly monitoring, with a diet plan and a target. Which is basically Slimming World, which is what I do anyway..” “….A pedometer that you bring back with you and you look at… that would be good”	Social influences Environmental context and resources Behavioral regulation

*NAFLD* nonalcoholic fatty liver disease.

## NAFLD DIAGNOSIS AND REFERRAL

### Knowledge

When interviewed patients were mostly concerned about the diagnostic process and the need for clear information about NAFLD, “I would have liked for it to have been explained how or why you get it, because they do not really.” It was emphasized that there was a lack of information about the risks associated with it and whether it is something to be concerned about.

### Beliefs about consequences

Diagnosis of NAFLD was reported by patients as being unexpected and usually a consequence of being investigated for something else. “It was only when I went for a visit, routinely, to the GP, for something completely different, that she said, ‘We have discovered that you have got this, and we need to do a blood test’.” Following diagnosis, patients reported being told by health care professionals that NAFLD was nothing to worry about, particularly when other comorbid conditions such as T2DM existed (i.e., that these were the priority). This meant that patients did not go in search for information about the condition themselves (e.g., from the internet) or feel the need to consider management approaches.

## NAFLD MANAGEMENT

### Knowledge

When asked about the management of NAFLD, patients found it difficult to provide their views on what they believed would be helpful due to not knowing exactly what NAFLD was and how it could be managed. They reported a lack of basic information about NAFLD when diagnosed, particularly in terms of whether it is something they should be concerned about, whether it could/should be managed, and if so how. “They tell you very little really. You know, you just get told that you’ve got fatty liver disease, but they’ll say a lot of people have fatty liver disease, it’s nothing to worry about”. This finding alone was considered a barrier to implementation of guidance in terms of NAFLD management. This was reinforced by health care professionals who believed that patients’ lack of knowledge may have prevented them from acting upon lifestyle behavior change advice given to them by members of the clinical team.

### Social influences

Patients reported being monitored for disease progression but emphasized a lack of information and support thereafter. Support was reported to consist of advice to lose weight and exercise more; however, this advice was rarely taken, particularly in situations where patients were told that NAFLD was nothing to worry about.

### Behavioral regulation

Patients found it difficult to provide suggestions to facilitate management of NAFLD in general, largely due to lack of information about what NAFLD is, how it progresses, and optimal management approaches. However, when lifestyle behavior change was mentioned, dietary plans and monitoring of diet and physical activity progress by a health care professional was reported as something that would be beneficial, “Yes, yes, like even if it was just monthly monitoring, with a diet plan and a target.” In terms of physical activity, patients suggested that a pedometer would be a useful tool to allow them to check and monitor their own progress.

A summary of barriers from the perspective of health care professionals and patients is presented in Table 3 with suggestions for intervention. These suggestions are based on our expert opinion as authors with expertise in the development of interventions in the context of health and lifestyle behavior change.

**Table 3:** Barriers to guideline implementation from the perspective of health care professionals and patients with suggestions for intervention

Barrier	Suggestion for intervention
Lack of awareness of guidance for the diagnosis and management of NAFLD	Raise awareness among primary and secondary care clinical teams of the availability of clinical guidelines
Variation in guideline adherence	Prompt routine use of clinical guidelines and identify training needs
Lack of knowledge of how to use validated tools to diagnosis NAFLD	Provide standardized training for clinical teams
Patients lack of knowledge of NAFLD and potential management approaches	Provide information to patients at the time of diagnosis to include a range of management options
Patients not following lifestyle advice	Emphasize the role of lifestyle behavior change for the management of NAFLD
Limited time during consultations to adequately target lifestyle behavior change	Provide training and tools to deliver brief intervention targeting lifestyle behavior change
Lack of lifestyle behavior change resources for use during consultations	Provide tools to target lifestyle behavior change for use during consultations
Lack of external lifestyle behavior change support services	Identification of and signposting to community lifestyle support services
Health care professionals’ lack of knowledge about NAFLD, including how it can be managed	Provide standardized training to clinical teams
Lack of knowledge and skills of health care professionals to effectively target lifestyle behavior change	Provide standardized training to clinical teams equipping team members with knowledge and skills to target lifestyle behavior change
Lack of lifestyle behavior change expertise in the clinical team	Provide training to all members of the multidisciplinary team to facilitate a consistent approach
Lack of support given to patients to make lifestyle changes	Provide training to clinical teams, including information about community lifestyle support services and tools that patients can use beyond the clinical consultation

*NAFLD* nonalcoholic fatty liver disease.

## DISCUSSION

We identified nine theoretical domains from the perspective of health care professionals that were considered either barriers or facilitators to guideline implementation for the diagnosis and management of NAFLD. In terms of diagnosis, they included beliefs about consequences and optimism. Overall, health care professionals believed that local guidance had improved NAFLD diagnosis rates (i.e., more patients with NAFLD were being identified) and referral rates (i.e., referrals to secondary and tertiary care were increasing) and that referrals were more informed and appropriate (i.e., specialist input was required in the majority of cases). Therefore, national and international guidance [18–20] had started to make a positive impact on practice behaviors. However, findings highlighted that there is a lack of awareness that guidelines exist and this has led to inconsistent referral behavior. The need to raise awareness about the availability of diagnostic tools and guidance was emphasized as well as the need for standardized training to ensure clinicians are using the guidance correctly (e.g., that they use validated tools correctly and consistently).

Seven theoretical domains were identified in the context of NAFLD management. These included beliefs about consequences, memory, attention, and decision processes, professional role and identity, knowledge, skills, environmental context and resources, and behavioral regulation. Beliefs about consequences was identified as most prominent in the context of NAFLD management, with the majority of health care professionals reporting that providing lifestyle advice would not make best use of time because patients rarely acted upon advice given. Monitoring was initially considered to be important to ensure that patients did not develop further liver problems; however, when explored further, it was acknowledged that this is not an optimal management approach in the context of lifestyle behavior change. Furthermore, many secondary care professionals indicated that it was not their role to address lifestyle behavior change. This emerged as a significant barrier to guideline implementation in the context of NAFLD management. Six of these nine domains were identified by a previous study that elicited primary health care professional’s perspectives on implementation of clinical guidelines for diabetes and hypertension [30], and five of these nine domains were identified by authors exploring adherence to multiple evidence-based indicators in primary care [31] suggesting that commonalities exist across conditions and care settings in the context of guideline implementation.

Four theoretical domains were identified from the perspective of patients. These were knowledge, beliefs about consequences, social influences, and behavioral regulation. Two domains (knowledge and beliefs about consequences) were identified in relation to NAFLD diagnosis. There was a consensus among patients that information provision at the time of diagnosis was lacking and management support thereafter was nonexistent. Any lifestyle advice provided was rarely acted upon by patients because they were advised that NAFLD was nothing to worry about.

In order to improve implementation of guidance for the diagnosis and management of NAFLD, the findings of this study highlight the need for interventions to improve the diagnostic process and subsequent management approach. We identified a number of theoretical domains that if targeted by an intervention have the potential to improve care delivery. Findings from patient interviews supported those of health care professionals, specifically the need for clear information at the time of NAFLD diagnosis for patients and a greater awareness among health care professionals of diagnostic criteria to ensure appropriate referrals are made to secondary and tertiary care. However, it emerged from interviews with primary health care professionals that they did not feel particularly knowledgeable about NAFLD and as such reported difficulties when communicating about the condition to patients, particularly around disease progression and management. This in part may explain why referrals to secondary and tertiary care were reported as inconsistent in terms of disease stage and why diagnostic and management advice was regularly sought from secondary and tertiary care professionals.

The theoretical domains knowledge and skills emerged as barriers to implementation of guidance from the perspective of primary and secondary health care professionals. Training provision to improve knowledge and skills in relation to diagnosis and lifestyle behavior change was frequently reported across interviews, although professional role and identity emerged as a barrier in some cases—that is, secondary/tertiary health care professionals in particular did not consider it as their role to target lifestyle behavior change in any significant depth during consultations. This could be an area for intervention. The suggestion for a dedicated member of the team to take on the role of working with patients to make lifestyle behavior changes, or a process for referring to external community lifestyle services, was favored. Therefore, environmental context and resources showed to be a significant facilitator in the context of NAFLD management and could be a target for intervention.

Patients consistently reported a desire to better understand their condition which in turn would motivate them to seek and engage with support to self-manage. Interviews with health care professionals emphasized that patients did not respond positively to management advice; however, without an understanding of their condition and the potential consequences of the diagnosis, it is understandable why patients are less likely to follow advice. Previous research has reported a similar finding in the context of engagement with a dietary intervention for NAFLD management [32].

The findings from this qualitative study supports a growing awareness of NAFLD among health care professionals in the community and the notion that the introduction of local guidelines [22] has prompted primary health care physicians to assess for NAFLD and refer to secondary care when appropriate. Primary health care professionals are in general requesting an increasing number of blood tests and encountering a rise in abnormal liver function tests and diagnoses of NAFLD [25]. Local guidelines appear to have been useful in standardizing diagnostic testing and have improved the appropriateness of referrals received by secondary care teams (i.e., increasingly patients are being triaged in primary care and only those at an indeterminate/high risk of advanced disease are being referred to secondary care for specialist opinion). Although the data highlight how guidance has impacted positively on health care professional behaviors, it appears that there are some primary health care professionals who are not currently following guidelines. This has been reported as lack of awareness of NAFLD and/or existence of the guidance and has been identified via this study as an area to target with intervention. Findings also highlight the importance of standardizing the pathway of care within individual medical practices to ensure consistency of care. In the UK, the recently published NICE guidelines [19] place emphasis on primary care physicians identifying NAFLD in higher-risk patient groups and assessing for advanced liver disease (i.e., liver fibrosis) prior to referring to a relevant specialist in hepatology. However, these guidelines rely on health care professionals being aware of and being knowledgeable about NAFLD and the findings of this qualitative study suggest that this is not always the case and that there is a clear training requirement.

Although there have been improvements in the diagnostic process for NAFLD, and the findings of this study provide support for this, management of NAFLD appears to be an ongoing issue. National and international guidelines recommend lifestyle modification/behavior change for the management of NAFLD [18–20]; however, these guidelines fail to provide specific details regarding how this should or could be achieved. Our findings suggest that current lifestyle management of patients with NAFLD largely consists of general advice to lose weight and exercise more with no specific information on how patients can achieve this or tailoring of information to individual patient needs or circumstances. We were able to explain this by identifying knowledge and skills in the context of lifestyle behavior change from the perspective of health care professionals, therefore identifying a training need in this regard.

## STRENGTHS AND LIMITATIONS

A strength of this study is that to the best of our knowledge, it is the first to report on barriers and enabling factors to guideline implementation in the context of NAFLD diagnosis and management with the aim of identifying targets for intervention. The findings report several issues with the diagnostic, referral, and lifestyle management procedures and practices but also provide suggestions from health care professionals and patients about how national and international guidelines could be implemented and thus care delivery improved.

Interview topic guides were developed with reference to published guidelines for the diagnosis and management of NAFLD and not based on the TDF. The advantage of this approach was that study participants (health care professionals and patients) were encouraged to respond to questions about diagnosis and management of NAFLD in relation to guidelines and not to questions specifically related to each theoretical domain (i.e., questions and responses were more focused and closely linked to practice). However, there was no response generated by the topic guide that could not be linked to a domain within the TDF, emphasizing the comprehensiveness of the framework used.

A further strength of this study was that both health care professionals and patients were interviewed. This allowed us to explore barriers and facilitators to guideline implementation from the perspectives of both groups and identify consensus. The approach was successful in this regard.

Interviews with health care professionals and patients were of relatively short duration which could be considered a limitation. However, it is likely that this reflects the lack of knowledge of primary health care professionals in particular and the lack of knowledge and awareness patients had in relation to their diagnosis. It is also possible that it reflects the little contact time patients have with health care professionals with regards to NAFLD and as such they had limited experiences to report. It was reassuring that data generated from patients supported data generated by health care professionals.

Health care professionals and patients were recruited to this study from a single region of Europe (North East England) with a high prevalence of NAFLD. Given the regional variation of service provision, it is possible that the views and experiences reported may not be representative nationally or internationally. However, steps were taken to ensure that a purposive sample of health care professionals and patients receiving treatment in primary, secondary, and tertiary care, from multiple providers, and health care professionals from specialist and generalist services were recruited. We believe that this approach increased the transferability of findings.

## CONCLUSIONS

Barriers to guideline implementation for NAFLD diagnosis included lack of awareness of local guidance and training of health care professionals to use validated tools and lack of information provision to patients. Barriers to NAFLD management included knowledge and skills of health care professionals to effectively support patients to make lifestyle changes, although professional role was also considered a barrier with many secondary health care professionals reporting lifestyle behavior change as not part of their role. A lack of resources and the belief that patients would fail to enact on lifestyle advice was also considered a barrier. Barriers to NAFLD management from the perspective of patients included lack of knowledge and awareness of what NAFLD is, whether it is progressive, and how it should or could be managed. Facilitators to implementation of guidance included awareness raising with health care professionals about the availability of local guidance for making a diagnosis and training on how to effectively use it. Information provision for patients at the time of diagnosis was believed to be a facilitator to engagement with NAFLD management. Facilitators to NAFLD management included training for clinical teams or, as a minimum, training of a designated individual within a team to target lifestyle behavior change in patients; provision of intervention resources to support lifestyle behavior change during consultations; online programmes to support patients to manage their condition outside of clinical appointments; and external lifestyle services to provide additional support to patients in the community. Patients were not able to provide a lot of information concerning NAFLD management due to lack of knowledge about what NAFLD is and how it can be managed but indicated that support to make lifestyle changes and tools to be able to monitor progress would be beneficial. The findings of this study will inform the development of an intervention for health care professionals and patients with an emphasis on guideline implementation and optimization of care delivery pathways for people with NAFLD.

## Supplementary Material

ibz080_Suppl_Supplementary_File_1

ibz080_Suppl_Supplementary_File_2

## References

[1] Younossi ZM, Koenig AB, Abdelatif D, Fazel Y, Henry L, Wymer M Global epidemiology of nonalcoholic fatty liver disease- Meta-analytic assessment of prevalence, incidence, and outcomes. Hepatology. 2016;64(1):73–84. 26707365 10.1002/hep.28431

[2] Younossi Z, Anstee QM, Marietti M, et al. Global burden of NAFLD and NASH: trends, predictions, risk factors and prevention. Nat Rev Gastroenterol Hepatol. 2018;15(1):11–20. 28930295 10.1038/nrgastro.2017.109

[3] Anstee QM, Targher G, Day CP Progression of NAFLD to diabetes mellitus, cardiovascular disease or cirrhosis. Nat Rev Gastroenterol Hepatol. 2013;10(6):330–344. 23507799 10.1038/nrgastro.2013.41

[4] Loria P, Adinolfi LE, Bellentani S, et al.; NAFLD Expert Committee of the Associazione Italiana per lo studio del Fegato Practice guidelines for the diagnosis and management of nonalcoholic fatty liver disease. A decalogue from the Italian Association for the Study of the Liver (AISF) expert committee. Dig Liver Dis. 2010;42(4):272–282. 20171943 10.1016/j.dld.2010.01.021

[5] Hallsworth K, Avery L, Trenell MI Targeting lifestyle behavior change in adults with NAFLD during a 20-min consultation: summary of the dietary and exercise literature. Curr Gastroenterol Rep. 2016;18(3):11. 26908279 10.1007/s11894-016-0485-1 PMC4764638

[6] Thoma C, Day CP, Trenell MI Lifestyle interventions for the treatment of non-alcoholic fatty liver disease in adults: a systematic review. J Hepatol. 2012;56(1):255–266. 21723839 10.1016/j.jhep.2011.06.010

[7] Petersen KF, Dufour S, Befroy D, Lehrke M, Hendler RE, Shulman GI Reversal of nonalcoholic hepatic steatosis, hepatic insulin resistance, and hyperglycemia by moderate weight reduction in patients with type 2 diabetes. Diabetes. 2005;54(3):603–608. 15734833 10.2337/diabetes.54.3.603 PMC2995496

[8] Kirk E, Reeds DN, Finck BN, et al. Dietary fat and carbohydrates differentially alter insulin sensitivity during caloric restriction. Gastroenterology. 2009;136(5):1552–1560. 19208352 10.1053/j.gastro.2009.01.048 PMC2677125

[9] Viljanen AP, Iozzo P, Borra R, et al. Effect of weight loss on liver free fatty acid uptake and hepatic insulin resistance. J Clin Endocrinol Metab. 2009;94(1):50–55. 18957499 10.1210/jc.2008-1689

[10] Sreenivasa Baba C, Alexander G, Kalyani B, et al. Effect of exercise and dietary modification on serum aminotransferase levels in patients with nonalcoholic steatohepatitis. J Gastroenterol Hepatol. 2006;21(1 Pt 1):191–198. 16706832 10.1111/j.1440-1746.2005.04233.x

[11] Johnson NA, Sachinwalla T, Walton DW, et al. Aerobic exercise training reduces hepatic and visceral lipids in obese individuals without weight loss. Hepatology. 2009;50(4):1105–1112. 19637289 10.1002/hep.23129

[12] Hallsworth K, Fattakhova G, Hollingsworth KG, et al. Resistance exercise reduces liver fat and its mediators in non-alcoholic fatty liver disease independent of weight loss. Gut. 2011;60(9):1278–1283. 21708823 10.1136/gut.2011.242073 PMC3152868

[13] Hallsworth K, Thoma C, Hollingsworth KG, et al. Modified high-intensity interval training reduces liver fat and improves cardiac function in non-alcoholic fatty liver disease: a randomized controlled trial. Clin Sci (Lond). 2015;129(12):1097–1105. 26265792 10.1042/CS20150308

[14] Oza N, Eguchi Y, Mizuta T, et al. A pilot trial of body weight reduction for nonalcoholic fatty liver disease with a home-based lifestyle modification intervention delivered in collaboration with interdisciplinary medical staff. J Gastroenterol. 2009;44(12):1203–1208. 19728009 10.1007/s00535-009-0115-x

[15] Albu JB, Heilbronn LK, Kelley DE, et al.; Look AHEAD Adipose Research Group Metabolic changes following a 1-year diet and exercise intervention in patients with type 2 diabetes. Diabetes. 2010;59(3):627–633. 20028945 10.2337/db09-1239 PMC2828653

[16] Promrat K, Kleiner DE, Niemeier HM, et al. Randomized controlled trial testing the effects of weight loss on nonalcoholic steatohepatitis. Hepatology. 2010;51(1):121–129. 19827166 10.1002/hep.23276 PMC2799538

[17] Vilar-Gomez E, Martinez-Perez Y, Calzadilla-Bertot L, et al. Weight loss through lifestyle modification significantly reduces features of nonalcoholic steatohepatitis. Gastroenterology. 2015;149(2):367–378.e5; quiz e14. 25865049 10.1053/j.gastro.2015.04.005

[18] European Association for the Study of the Liver (EASL); European Association for the Study of Diabetes (EASD); and European Association for the Study of Obesity (EASO). EASL–EASD–EASO Clinical Practice Guidelines for the management of non-alcoholic fatty liver disease. J Hepatol., 2016;64(6):1388–1402. 27062661 10.1016/j.jhep.2015.11.004

[19] National Institute for Clinical Excellence. Non-Alcoholic Fatty Liver Disease (NAFLD): Assessment and Management (NG49). National Institute for Health and Clinical Excellence (NICE), London 2016. 27441333

[20] Chalasani N, Younossi Z, Lavine JE, et al. The diagnosis and management of nonalcoholic fatty liver disease: Practice guidance from the American Association for the Study of Liver Diseases. Hepatology. 2018;67(1):328–357. 28714183 10.1002/hep.29367

[21] Avery L, Exley C, McPherson S, Trenell MI, Anstee QM, Hallsworth K Lifestyle behavior change in patients with nonalcoholic fatty liver disease: A qualitative study of clinical practice. Clin Gastroenterol Hepatol. 2017;15(12):1968–1971. 28624648 10.1016/j.cgh.2017.06.011

[22] Hudson M, McPherson S, on behalf of the North East and North Cumbria Hepatology Network Clinical Guidelines: Revised Newcastle, North Tyneside and Northumberland Guidelines for the Management of Adults with Asymptomatic Liver Function Abnormalities. 2014 Available at http://api.gp.northumbria.nhs.uk/uploads/downloads/RMS%20Guidelines/-%20Gastroenterology/tmpManagement_of_Adults_with_Asymptomatic_Liver_Function_Abnormalities.pdf. Accessed October 18, 2018.

[23] McPherson S, Hardy T, Dufour JF, et al. Age as a confounding factor for the accurate non-invasive diagnosis of advanced NAFLD fibrosis. Am J Gastroenterol. 2017;112(5):740–751. 27725647 10.1038/ajg.2016.453 PMC5418560

[24] Tsochatzis EA, Newsome PN Non-alcoholic fatty liver disease and the interface between primary and secondary care. Lancet Gastroenterol Hepatol. 2018;3(7):509–517. 29893235 10.1016/S2468-1253(18)30077-3

[25] Palinkas LA, Horwitz SM, Green CA, Wisdom JP, Duan N, Hoagwood K Purposeful sampling for qualitative data collection and analysis in mixed method implementation research. Adm Policy Ment Health. 2015;42(5):533–544. 24193818 10.1007/s10488-013-0528-y PMC4012002

[26] Morse JM Critical analysis of strategies for determining rigor in qualitative inquiry. Qual Health Res. 2015;25(9):1212–1222. 26184336 10.1177/1049732315588501

[27] Cane J, O’Connor D, Michie S Validation of the theoretical domains framework for use in behaviour change and implementation research. Implement Sci. 2012;7:37. 22530986 10.1186/1748-5908-7-37 PMC3483008

[28] Atkins L, Francis J, Islam R, et al. A guide to using the Theoretical Domains Framework of behaviour change to investigate implementation problems. Implement Sci. 2017;12(1):77. 28637486 10.1186/s13012-017-0605-9 PMC5480145

[29] Francis JJ, Johnston M, Robertson C, et al. What is an adequate sample size? Operationalising data saturation for theory-based interview studies. Psychol Health. 2010;25(10):1229–1245. 20204937 10.1080/08870440903194015

[30] Chimeddamba O, Peeters A, Ayton D, Tumenjargal E, Sodov S, Joyce C Implementation of clinical guidelines on diabetes and hypertension in urban Mongolia: a qualitative study of primary care providers’ perspectives and experiences. Implement Sci. 2015;10:112. 26259569 10.1186/s13012-015-0307-0 PMC4531849

[31] Lawton R, Heyhoe J, Louch G, et al.; ASPIRE programme Using the Theoretical Domains Framework (TDF) to understand adherence to multiple evidence-based indicators in primary care: a qualitative study. Implement Sci. 2016;11:113. 27502590 10.1186/s13012-016-0479-2 PMC4977705

[32] Haigh L, Bremner S, Houghton D, et al. Effective dietary interventions for non-alcoholic fatty liver disease: barriers and facilitators to adoption of a Mediterranean diet in a northern European patient population. Clin Gastroenterol Hepatol. 2017;s1542-3565(18)31210-2. Epub ahead of print.

