# Supplementary material for: Using the theoretical domains framework to identify barriers and enabling factors to implementation of guidance for the diagnosis and management of nonalcoholic fatty liver disease: a qualitative study
Source: Transl Behav Med. 2019 May 23;10(4):1016–30. doi: 10.1093/tbm/ibz080 (PMC7543077; doi:10.1093/tbm/ibz080)
Supplement: ibz080_Suppl_Supplementary_File_1 [file ibz080_suppl_supplementary_file_1.pdf]

Development of the first evidence based lifestyle service pathway for people with non-alcoholic fatty liver disease (NAFLD)

Phase 1: Exploring the current service pathway

Interview Schedule (Semi-structured)

Patients

1. Lead up to diagnosis of NAFLD: why did you go to see the doctor/nurse/healthcare professional?
2. Who diagnosed you (i.e. which healthcare professional NOT a named person)? Where (e.g. GP surgery, hospital)?
3. When/how many years ago were you diagnosed?
4. How were you diagnosed (including investigations undertaken)?
5. Was the diagnosis explained/did you understand the diagnosis? How would you explain your condition to someone else?
6. What happened then? (to include management/advice received and any follow up)
7. Which healthcare professionals were involved in your care?
8. Could your care have been improved in any way? If so how?
9. What would the "ideal" service look like? Who would deliver this service? Where would it be delivered?
10. Have you ever taken part in any lifestyle interventions as part of your medical care for other conditions? (e.g. DESMOND, Exercise on Referral, Weight Management) What was your experience like?
11. Would any other input have been beneficial in the NAFLD care pathway?

Healthcare Professionals

1. Why are patients with suspected NAFLD normally referred to you? In Primary Care, what normally brings these patients to see you?
2. How are patients diagnosed with NAFLD?
3. Which tools/investigations are routinely used in the diagnosis of NAFLD?
4. What happens after a diagnosis of NAFLD is made (in terms of management/advice given and if/when follow up is required)?
5. For GPs: when do you make a referral to Secondary Care?
6. For Secondary Care Physicians: when do you refer back to Primary Care?
7. When/why are referrals made to other services within/outside the NHS?
8. Could the NAFLD care pathway be improved? If so how?
9. What would the "ideal" service look like? Who would deliver this service? Where would it be delivered?
10. Would any tools be useful to support the lifestyle element of patient care?
11. Have you ever received any specific training with regards to lifestyle behavior change in general or motivational interviewing?
